# The humanistic and economic burden of chronic wounds: a protocol for a systematic review

**DOI:** 10.1186/s13643-016-0400-8

**Published:** 2017-01-24

**Authors:** Krister Järbrink, Gao Ni, Henrik Sönnergren, Artur Schmidtchen, Caroline Pang, Ram Bajpai, Josip Car

**Affiliations:** 1 Centre for Population Health Sciences (CePHaS), Lee Kong Chian School of Medicine, Nanyang Technological University, 59 Nanyang Drive, Experimental Medicine Building, Singapore, 636921 Singapore; 2 Department of Dermatology, Skåne University Hospital, Lasarettsgatan 15, 221 85 Lund, Sweden; 3 Dermatology and Innate Immunity Laboratory, Lee Kong Chian School of Medicine, Nanyang Technological University, 59 Nanyang Drive, Experimental Medicine Building, Singapore, 636921 Singapore; 4 Medical Library, Lee Kong Chian School of Medicine, 11 Mandalay Road, Singapore, 308232 Singapore; 5 Global eHealth Unit, Department of Primary Care and Public Health, School of Public Health, Imperial College London, 3rd Floor Reynolds Building, St Dunstan’s Road, London, W6 8RP UK

**Keywords:** Chronic wounds, Hard-to-heal ulcers, Quality of life, Health-related quality of life, Quality-adjusted life years, Cost of illness, Costs and cost analysis, Economics

## Abstract

**Background:**

Chronic non-healing wounds present a substantial economic burden to healthcare system; significant reductions in quality of life for those affected, and precede often serious events such as limp amputations or even premature deaths. This burden is also likely to increase with a larger proportion of elderly and increasing prevalence of life-style diseases such as obesity and diabetes. Reviews of the evidence on the burden of illness associated with chronic wounds have not been comprehensive in scope and have not provided an assessment of the distribution of the health care costs across categories of resource use.

**Methods/design:**

This study is a systematic review of multiple databases for studies on adult patients with chronic wounds and with the primary objective to assess the impact on health-related quality of life by category of ulcers, and associated direct and indirect costs. Eligible studies will primary be empirical studies evaluating, describing or comparing measurement of quality of life and economic impact. Two reviewers will independently screen titles and abstracts and select studies involving adults with chronic wounds. These investigators will also independently extract data using a pre-designed data extraction form. Differences in applied methodologies and uncertainties will clearly be accounted for. Conservative valuations of costs and impact on health-related quality of life will be prioritised. Variations that may depend on age distribution, the categorisation of ulcer, healthcare system etc. will be described clearly.

**Discussion:**

The proposed systematic review will yield a comprehensive assessment of the humanistic and economic burden of chronic wounds in an adult population. A better understanding of the humanistic and economic burden of chronic wounds is essential for policy and planning purposes, to monitor trends in disease burden and not at least in order to estimate the real-world cost-effectiveness of new treatments and therapies.

**Systematic review registration:**

PROSPERO CRD42016037496

**Electronic supplementary material:**

The online version of this article (doi:10.1186/s13643-016-0400-8) contains supplementary material, which is available to authorized users.

## Background

A chronic wound can be defined as one that has failed to proceed through an orderly and timely reparative process to produce anatomic and functional integrity within a period of 3 months or that has proceeded through the repair process without establishing a sustained and anatomic and functional result [1, 2]. The nomenclature is far from agreed upon, and these wounds are sometimes referred to as hard-to-heal or difficult to heal wounds/ulcers, and the time span required for chronicity has been defined in the range from 4 weeks up to more than 3 months [2–4]. Based on the causative aetiologies, the Wound Healing Society classifies chronic wounds into four categories: pressure ulcers, diabetic ulcers, venous ulcers and arterial insufficiency ulcers [5]. Older people are the highest risk group for chronic wounds given that wound repair slows down as the body ages [6, 7] and incidences of cardiovascular disease and diabetes (which increase the incidence of chronic wounds) also increase with age [8]. The basic biology and the influence of age-associated changes on wound healing are poorly understood, and there are numerous research questions still to be answered [7].

Wounds are typically managed as a co-morbidity of other conditions, limiting the impact of efforts to overcome the growing challenge they represent. Clinicians often lack specialised training in the diagnosis and treatment of wounds because it is not a defined specialisation and different specialists such as dermatologists, podiatrists, endocrinologists, vascular surgeons and geriatricians, maybe involved in the care to a different extent in different healthcare systems. From a policy perspective, this fragmented responsibility has led to a lack of prioritisation of resources and capabilities around wound care, and in a clinical sense has produced inconsistent treatments, prolonged healing and an uncoordinated approach to prevention [9]. It is a ‘silent epidemic’ [10], and this has often resulted in inadequate planning and poor implementation of prevention, treatment and management strategies. Consequently, wound research is an important but neglected field. The lack of research has compromised innovation in new therapies, diagnostics, clinical practices and procedures. For example, the scientific literature highlights the lack of knowledge on the biological processes associated with wound healing and limited evidence for effective pathways of clinical care and therapies for prevention and healing [11].

Although wound care is a multibillion-dollar worldwide problem, that only in the USA affect 5.7 million people (~2% of population) at an annual cost of US$20 billion [12]. A UK report suggested that treatment and care of chronic wounds accounts for 3% of total healthcare expenditure in developed countries [13], but this is probably somewhat low because a more recent study from Wales using estimates of prevalence and cost from routine NHS data concludes that chronic wounds have a prevalence of 6% and consume at least 5.5% of NHS expenditure [14].Chronic non-healing wounds would thereby impose an immense financial burden to the society, not only through an economic burden on the health care system but also through a reduction in productivity [15].

Chronic wounds may require several years to heal, and some remain unhealed for decades. During this time, patients can experience severe pain, significant emotional and physical distress, reduced mobility and social isolation [16]. Studies have also shown that chronic wounds not only cause severe emotional and physical trauma to the patient but also to their families [17]. Chronic wounds may also result in disabilities after all available therapeutic interventions have been exhausted, and an amputation is necessary. Ulcers precede 85% of all amputations [18] while diabetic ulcer is the reason for 70% of all lower limb amputations. Globally, there is an amputation every 30 s due to a non-healing diabetic ulcer [19]. The 5-year mortality rate following amputation is reported to be between 40 and 70% [20] and is higher among patients with major amputation [21]. Unfortunately, events such as infections, amputations and death as a consequence of a wound are all too common and may be avoidable with accurate diagnosis and early appropriate treatment.

The ageing populations in many parts of the world and the increasing incidence of chronic diseases make the requirement for improved wound care urgent and critical. However, to enable effective implementation, there needs to be greater awareness about the increasing clinical challenge that wounds present, and methodology needs to be developed to ascertain and ensure that cost-effective clinical practices are employed. Currently, there is a lack of research on the humanistic and economic burden of chronic wounds and there is consequently a need to comprehensively quantify the burden of chronic wounds to the individual and healthcare system.

An improved knowledge would equip decision makers with a tool for evaluating therapies and an increased understanding of what drives a cost-effective wound care. Accurate and robust measurement of disease burden is therefore imperative for the future planning of healthcare services and optimisation of clinical pathways. The development of new knowledge and innovative technologies, and the widespread adoption of these developments into best practice clinical pathways is also required if a more effective wound care is to be achieved. There are great challenges ahead. A systematic review published in 2014 has shown that few cost-effectiveness studies exist to guide decision makers regarding guideline-based or strategic interventions for chronic wounds [22]. However, existing evidence point in the direction that more specialised and intense wound care is cost-effective and not rarely cost-saving [23]. Moreover, a Danish study has argued that by adopting a national strategy based on best practice guidelines, it may be possible to reduce the costs for wound management with up to 30% [24].

A systematic review of the humanistic and economic burden of chronic wounds is essential for policy and planning purposes, to monitor trends in disease burden and not at least in order to estimate the real-world cost-effectiveness of new treatments and therapies. The increasing number of elderly and the increasing prevalence of lifestyle diseases such as obesity and diabetes will result in that the burden of chronic wounds will be considerably larger if nothing is made. New treatments, therapies and preventive interventions require cost-effectiveness studies that would benefit hugely from burden-of-disease estimates.

The systematic review’s outcome consists of data on quality of life and cost consequences for pressure ulcers, diabetic ulcers, venous ulcers and arterial insufficiency ulcers. Quality of life or health-related quality of life (HRQoL) is a patient-reported outcome (PRO) which means that it is a report of the status of a patient’s health condition that comes directly from the patient, without interpretation of the patient’s responses by a physician or anyone else. Common HRQoL measures include EuroQol (EQ-5D), Short-Form 36 (SF-36), health utilities index (HUI) and the visual analogue scale (VAS).

The economic definition of cost is the value of opportunity forgone, strictly the best opportunity forgone, as a result of engaging resources in an activity, in this case, the care of chronic wounds. Costs do also arise without the exchange of money and extends beyond those falling on the healthcare service alone, e.g. patients themselves and families, caregivers and communities.

The overall aim of this paper is to present a transparent process for how the information will be collected on the humanistic and economic burden of chronic wounds and related complications. This will include the key research questions that this review will address description of systematic literature search strategies, criteria for inclusion or exclusion of studies, description of coding procedures, study quality measures and statistical procedures for the quantitative analysis of data from eligible studies.

## Methods/design

### Protocol

The methods for this systematic review have been developed according to the recommendations from the Preferred Reporting Items for Systematic Review and Meta-Analysis Protocols (PRISMA-P) 2015 statement [25]. This systematic review protocol has been registered in the International Prospective Register of Systematic reviews (PROSPERO): CRD42016037496). A PRISMA-P file is attached (see Additional file 1).

### Research questions

The overall objective is to determine the burden of chronic wounds and related complications. The focus is on chronic wounds in the categories of pressure ulcers, diabetic ulcers, venous ulcers and arterial insufficiency ulcers.

Specific review questions are as follows:

- What is the impact on the patient’s and carer’s health-related quality of life of chronic wounds in the categories of pressure ulcers, diabetic ulcers, venous ulcers and arterial insufficiency ulcers?
- What is the reported economic impact of chronic wounds in the categories of pressure ulcers, diabetic ulcers, venous ulcers and arterial insufficiency ulcers?

### Eligibility criteria

#### Population

The population of interest will include adult patients 18 years of age and older with pressure ulcers, diabetic ulcers, venous ulcers and arterial ulcers. Patients with chronic wounds resulting from non-preventable surgical wounds and wounds from skin tumours will be excluded.

#### Outcome

- Impact on HRQoL for patients and carers of defined chronic wounds and complications measured either over different generic domains (e.g. EQ-5D, SF-36, HUI) or by a single-index health utility measure (e.g. time trade-off (TTO), standard gamble (SG), visual analogue scale (VAS)).
- Measured and valued cost consequences of defined chronic wounds and specified complications thereof. Measured and valued cost consequences could be either incidence or prevalence based.

#### Study design

Studies will be restricted by design and observational studies, cross-sectional studies, cohort studies, case-control studies, single arm and systematic review/meta-analyses will be included.

Non-research letters and editorials, seminar reviews and case studies/series reporting cases will be excluded. Studies reporting health-related quality of life will be restricted to include studies that measures patient-reported generic HRQoL and studies that measures disease specific HRQoL will consequently be excluded.

Randomised controlled trials will be excluded based on methodological inappropriateness of research design for the type of questions to be answered. Economic modelling studies will be excluded as they present data for costs or cost-effectiveness related to specific interventions under investigation.

### Search strategy

We will begin by developing a comprehensive database containing empirical studies evaluating, describing or comparing measurement of quality of life and/or the economic impact of chronic wounds (including hard-to-heal wound/ulcers) and complications thereof in general populations. A systematic search of MEDLINE (Ovid), EMBASE (Ovid), EBM Reviews and Cochrane (Ovid), Cumulative Index to Nursing and Allied Health Literature (CINAHL) (EBSCO), PsycINFO (EBSCO) and Global Health (EBSCO) will be undertaken. As we are primarily interested in the contemporary literature on this topic, we will examine publications from January 2000 through December 2015.

To construct a comprehensive set of possible search terms, we list indexing terms (for example, subject headings and subheadings, publication types) and text words used to describe concept clusters (single words or phrases that may appear in titles or abstracts, both in full and in various truncations). For instance, we search pressure ulcer with its keyword “pressure ulcer” with all heading and subheadings, and then we search alternative keywords “pressure sore” or “bed ulcer” or “bed sore” appearing in the title or abstract in the form of full or truncations. We sought further terms from clinicians and librarians, and from published strategies from other groups. The search strategy was developed by the research team in collaboration with an experienced medical research librarian at the Lee Kong Chian School of Medicine. Additional comments and suggestions were also received from an experienced librarian at the Karolinska Institutet and incorporated. The search was revised, as necessary, and the final MEDLINE search is presented in Additional file 2. The MEDLINE strategy will be adapted to the syntax and subject headings of other databases.

Searches will be limited to peer-reviewed full text articles in English language and letters, abstracts, and editorials are to be excluded. There will be no geographical limitation on the included studies. Contact with authors for further information will be made when necessary. The reference lists of all identified articles from January 2000 and beyond will also be searched for any additional sources of information. An additional search will be made using the newly introduced term “pressure injury” [26] and relevant articles added.

### Study records

#### Data management

We will implement the search strategies and import all references identified to EndNote. The search results from the different electronic databases will be combined in a single EndNote library and we will remove duplicate records of the same reports.

### Selection process

Two reviewers will independently screen titles and abstracts against eligibility criteria to identify potentially included studies. Specifically, titles and abstracts are included if they indicate that it was a population-based or institutional-based study reporting any relevant information on the humanistic or economic burden of chronic wounds and/or complications thereof. In the next phase, we will retrieve full-text copies of those articles deemed potentially relevant. Two reviewers will independently assess the full text of the retrieved articles for compliance with our eligibility criteria. Discrepancies between the two reviewers’ judgement will be resolved by discussion or by the involvement of a third reviewer. Studies, which appeared to be relevant, but excluded at this stage will be listed in the table “Characteristics of excluded studies”, where a reason for exclusion will be noted. Two reviewers will verify the final list of included studies. A PRISMA flow diagram of the study selection procedure will be prepared to provide an overview of the decisions that are made in the data collection process.

### Data collection process

Two reviewers will independently extract and manage the data for each of the included studies using an electronic data extraction form. We will pilot the data extraction form and amend it according to feedback received from a panel of experienced colleagues. We plan to contact study authors in case of any unclear or missing information. Disagreements between review authors will be resolved by discussion. A third review author will act as an arbiter in case disagreements cannot be resolved.

### Data items

Data will be extracted on the following:

1. Publication details: title, journal, author, year, city and country, in which the study was conducted, type of publication, and source of funding.
2. Design: type of study (observational studies, cross-sectional, cohort, case-control, single arm, systemic review/meta-analyses); aims of study, method of data collection, response rate, recruitment methods, eligibility (inclusion and exclusion criteria).
3. Study participant details: number of persons interviewed or surveyed, population characteristics including age, gender, ethnicity, demographic information, diagnostics, ulcer specifications, complications.
4. Data for outcome measures. Humanistic burden: utility score patient, utility score carer, utility instrument/method applied, scoring algorithm, mode of administration, utility control patient, utility control carer, limitations in the measurement of utility. Economic burden: cost currency, cost sources, whether prevalence-based COI or incidence-based COI, recall period, mode of administration, discounting (incidence-based), cost inpatient care, cost outpatient specialist care, cost primary care, cost pharmaceuticals, cost community care, cost aid/utensils, cost productivity losses, productivity loss, method of estimation, productivity loss, per cent of full working time, cost informal care, method of estimation, informal care per cent of full working time, limitations/needed improvements.

The primary outcome will be impact on HRQoL by category of ulcers and associated direct and indirect costs. Differences in applied methodologies, ulcer specifications and other uncertainties will clearly be accounted for. Conservative valuations of costs and impact on HRQoL will be prioritised. Variations that may depend on age distribution, the categorisation of ulcer, study design etc. will be described clearly. Secondary outcomes will be the impact on carers’ HRQoL, dropping out and the reasons for that.

### Risk of bias in individual studies

As a variety of study types and data sources are likely to be eligible from the literature, information will be recorded on the appropriateness of the particular study design to estimate relevant parameters and the representativeness of the population. There are many sources for methodological biases starting from research question itself, selection bias, information bias, confounding and overall quality of a study. Therefore, studies reporting health-related quality of life will be critically appraised using the Joanna Briggs Institute critical appraisal tool which is developed for prevalence data but also suitable for quality of life data that is collected with validated and well-recognised instruments [27]. Studies reporting the economic burden will be appraised using the Drummond and Jefferson checklist [28].

The quality assessment will be independently conducted by two reviewers. Any discrepancies will be discussed, and if required, a third reviewer will be consulted. Publication bias and selective reporting will be dealt with by critically assessing study findings, plots will be made of outcome variables against sample size [25] and advice will be taken from GRADE guidelines No. 5 [29].

### Data synthesis

For studies on quality-of-life, a hierarchical linear model will be used to perform a meta-regression in which quality of life is the independent variable while elicitation method, respondents’ characteristics, instrument, category of wound and wound duration will be tested as explanatory variables.

Meta-analysis of international cost data is difficult because of transferability due to geographic origin. However, all costs will be presented in the reference year of 2015 in US dollars using the consumer price index (CPI) for each country [30] and the year 2015 purchasing power parity (PPP) conversion factor [31, 32] to allow for greater transparency and comparability across studies.

### Statistical analysis

Descriptive analysis will be performed based on the collective information from the studies and summarised into tables. The forest plots will be made for quality of life and cost data indicators with their 95% confidence intervals (C.I.s) to visualise publication bias. Further, the subgroup analysis and sensitivity analysis will also be performed to address the variability across studies. If the collective evidence of studies allowed, the study outcomes will be further stratified according the background outcome variables. Choice of meta-analysis will also be based on the homogeneous group of studies. For studies on quality of life (QOL), a hierarchical linear model will be used to perform a meta-regression in which quality of life is the independent variable while elicitation method, respondents, instrument etc. could be explanatory variables. Parametric meta-analyses may be considered for cost data based on the number of the eligible studies. The decision of fixed effect model will be based on pooled data using the appropriate statistical method and the choice of random-effects model will be based on clinical and methodological diversity across the included studies

### Summary

This systematic review will be performed to critically examine the world’s relevant literature on the humanistic and economic burden of chronic wounds. Specifically, we aim to identify and report the estimated humanistic and economic burden of chronic wounds in the categories of pressure ulcers, diabetic ulcers, venous ulcers and arterial insufficiency ulcers and related complications for different settings and subgroups. Understanding the humanistic and economic impact burden of different categories of complications among patients with chronic wounds could inform policy makers on the cost effectiveness of implementing early screening and other prevention or treatment efforts. Also, quantifying the humanistic and economic burden of chronic wounds will help guide decision-making for the allocation of scarce healthcare resources and funding. A further finding of this systematic review will be the methodological assessment of the published literature. The findings of this review will also be compared with other similar published reviews. Finally, conclusions will be drawn from this systematic review highlighting the burden of chronic wounds, methods of estimation and settings and their correlates. Limitations of the studies will be discussed in detail. Implications of the review as well as suggestions for future research will also be provided.

## Additional files

Additional file 1: PRISMA-P checklist. (DOC 83 kb)
Additional file 2: MEDLINE search strategy. (DOCX 32 kb)

## Abbreviations

CINAHL: Cumulative Index to Nursing and Allied Health Literature
EQ-5D: EuroQol-5D
GRADE: Grading of Recommendations Assessment Development and Evaluation
HRQoL: Health-related quality of life
HUI: Health utilities index
PRISMA-P: Preferred reporting items for systematic reviews and meta-analyses protocols
PROSPERO: International prospective register of systematic reviews
SF-36: Short-Form 36
SG: Standard gamble
TTO: Time trade-off
VAS: Visual analogue scale
