# Supplementary material for: The humanistic and economic burden of chronic wounds: a protocol for a systematic review
Source: Syst Rev. 2017 Jan 24;6:15. doi: 10.1186/s13643-016-0400-8 (PMC5259833; doi:10.1186/s13643-016-0400-8)
Supplement: Additional file 2: — MEDLINE search strategy. (DOCX 32 kb) [file 13643_2016_400_MOESM2_ESM.docx]

**MEDLINE (Ovid) search strategy**

1. exp Pressure Ulcer/

2. (pressur$ adj3 ulcer$).tw.

3. (pressur$ adj3 sore$).tw.

4. exp decubitus ulcer/

5. (decubitus adj3 ulcer$).tw.

6. (decubitus adj3 sore$).tw.

7. (bed adj3 ulcer$).tw.

8. (bed adj3 sore$).tw.

9. bedsore$.tw.

10. (arterial adj3 ulcer$).tw.

11. (ischemic adj3 ulcer$).tw.

12. (neuropathic adj3 ulcer$).tw.

13. exp Varicose Ulcer/

14. (vascul$ adj3 ulcer$).tw.

15. (varicose adj3 ulcer$).tw.

16. exp venous ulcer/

17. (venous adj3 ulcer$).tw.

18. exp stasis ulcer/

19. (stasis adj3 ulcer$).tw.

20. exp Skin Ulcer/

21. (skin adj3 ulcer$).tw.

22. exp Foot Ulcer/

23. (foot adj3 ulcer$).tw.

24. (diabetic adj3 foot).tw.

25. (diabetic adj3 feet).tw.

26. (diabetic adj3 ulcer).tw.

27. exp Leg Ulcer/

28. (leg adj3 ulcer$).tw.

29. (chronic adj3 wound$).tw.

30. (chronic adj3 sore$).tw.

31. (chronic adj3 ulcer$).tw.

32. (crural adj3 ulcer$).tw.

33. (ulcus adj3 cruris).tw.

34. non-healing wound$.tw.

35. hard to heal.mp.

36. cost$.ti,ab,kf.

37. exp costs/ and cost analysis/

38. cost effective$.tw.

39. 36 or 37 or 38

40. (health care rationing or health priorities or medical savings account$ or resource allocation).ti,ab,kf.

41. (health care rationing or healthcare rationing or health priorities or medical savings account$ or resource allocation).ti,ab,kf.

42. (budget$ or cost$ or econom$ or expenditure$ or fee$ or financ$ or health resource or money or pharmacoeconomic$ or socioeconomic$).ti,ab,kf.

43. (budget$ or cost$ or econom$ or expenditure$ or fee$1 or financ$ or health resource or money or pharmacoeconomic$ or socioeconomic$).ti,ab,kf.

44. (budget$ or cost$ or econom$ or expenditure$ or financ$ or fiscal or funding or pharmacoeconomic$ or socioeconomic$ or price or prices or pricing).tw.

45. ((value adj3 money) or (burden adj3 (disease$ or illness$))).tw.

46. 40 or 41 or 42 or 43 or 44 or 45

47. 39 or 46

48. exp quality of life/

49. (quality of life or life satisfaction or quality of well being or quality of living or standard of living).ti,ab,kf.

50. exp value of life/

51. (value of life or respect for life or cost of life or potency of life or purpose of life or meaning of life or appreciation of life).ti,ab,kf.

52. exp quality adjusted life year/

53. quality adjusted life.ti,ab,kf.

54. (qaly$ or qald$ or qale$ or qtime$).ti,ab,kf.

55. disability adjusted life.ti,ab,kf.

56. daly$.ti,ab,kf.

57. (sf36 or sf 36 or short form 36 or shortform 36 or sf thirtysix or sf thirty six or shortform thirtysix or shortform thirty six or short form thirtysix or short form thirty six).ti,ab,kf.

58. (sf12 or sf 12 or short form 12 or shortform 12 or sf twelve or sftwelve or shortform twelve or short form twelve).ti,ab,kf.

59. (sf6 or sf 6 or SF 6 or short form 6 or shortform 6 or sf six or sfsix or shortform six or short form six).ti,ab,kf.

60. (sf16 or sf 16 or short form 16 or shortform 16 or sf sixteen or sfsixteen or shortform sixteen or short form sixteen).ti,ab,kf.

61. (sf20 or sf 20 or short form 20 or shortform 20 or sf twenty or sftwenty or shortform twenty or short form twenty).ti,ab,kf.

62. (euroqol or "euro qol" or "eq5d" or "eq 5d").ti,ab,kf.

63. (hql or hqol or "h qol" or hrqol or "hr qol").ti,ab,kf.

64. ("hye" or "hyes").ti,ab,kf.

65. health$ year$ equivalent$.ti,ab,kf.

66. (hui or hui1 or hui2 or hui3).ti,ab,kf.

67. disutil$.ti,ab,kf.

68. quality of well being.ti,ab,kf.

69. quality of wellbeing.ti,ab,kf.

70. qwb.ti,ab,kf.

71. willingness to pay.ti,ab,kf.

72. standard gamble*.ti,ab,kf.

73. time trade off.ti,ab,kf.

74. time tradeoff.ti,ab,kf.

75. tto.ti,ab.

76. (index adj2 well being).ti,ab,kf.

77. (quality adj2 well being).ti,ab,kf.

78. qwb.tw.

79. (health adj3 util$ adj ind$).ti,ab,kf.

80. health utilities index.ti,ab,kf.

81. or/48-80

82. 47 or 81

83. or/1-35

84. 82 and 83

85. limit 84 to (english language and humans and yr="2000 - 2015" and “all adult (19 plus years)”)
